# SWI and phase imaging reveal intracranial calcifications in the P301L mouse model of human tauopathy

**DOI:** 10.1007/s10334-020-00855-3

**Published:** 2020-05-28

**Authors:** Ruiqing Ni, Yvette Zarb, Gisela A. Kuhn, Ralph Müller, Yankey Yundung, Roger M. Nitsch, Luka Kulic, Annika Keller, Jan Klohs

**Affiliations:** 1 Institute for Biomedical Engineering, University of Zurich and ETH Zurich, Vladimir-Prelog-Weg 4, Wolfgang-Pauli-Strasse 27, 8093 Zurich, Switzerland; 2 Zurich Neuroscience Center, University of Zurich, Zurich, Switzerland; 3 Department of Neurosurgery, Zurich University Hospital, Sternwartstrasse 6 (G3), 8091 Zurich, Switzerland; 4 Department of Health Sciences and Technology, Institute for Biomechanics, ETH Zurich, Leopold-​Ruzicka-Weg 4, 8093 Zurich, Switzerland; 5 Institute for Regenerative Medicine, University of Zurich, Wagistrasse 12, 8952 Schlieren, Switzerland

**Keywords:** Susceptibility weighted imaging, Phase imaging, Alzheimer’s disease, Calcification, Tauopathy, Transgenic mouse model

## Abstract

**Objective:**

Brain calcifications are associated with several neurodegenerative diseases. Here, we describe the occurrence of intracranial calcifications as a new phenotype in transgenic P301L mice overexpressing four repeat tau, a model of human tauopathy.

**Materials and methods:**

Thirty-six P301L mice (Thy1.2) and ten age-matched non-transgenic littermates of different ages were assessed. Gradient echo data were acquired in vivo and ex vivo at 7 T and 9.4 T for susceptibility-weighted imaging (SWI) and phase imaging. In addition, ex vivo micro-computed tomography (μCT) was performed. Histochemistry and immunohistochemistry were used to investigate the nature of the imaging lesions.

**Results:**

SW images revealed regional hypointensities in the hippocampus, cortex, caudate nucleus, and thalamus of P301L mice, which in corresponding phase images indicated diamagnetic lesions. Concomitantly, µCT detected hyperdense lesions, though fewer lesions were observed compared to MRI. Diamagnetic susceptibility lesions in the hippocampus increased with age. The immunochemical staining of brain sections revealed osteocalcin-positive deposits. Furthermore, intra-neuronal and vessel-associated osteocalcin-containing nodules co-localized with phosphorylated-tau (AT8 and AT100) in the hippocampus, while vascular osteocalcin-containing nodules were detected in the thalamus in the absence of phosphorylated-tau deposition.

**Discussion:**

SWI and phase imaging sensitively detected intracranial calcifications in the P301L mouse model of human tauopathy.

**Electronic supplementary material:**

The online version of this article (10.1007/s10334-020-00855-3) contains supplementary material, which is available to authorized users.

## Introduction

Brain calcification is the most common incidental finding of patients undergoing neuroimaging [1, 2]. Computed tomography (CT) is considered the non-invasive gold standard for the identification of intracranial calcification because it allows discriminating calcification from other sources of focal contrast (hemorrhagic lesions or infarctions). It is estimated that 20% of aged individuals have brain calcifications, where they are particularly detected in the pineal gland and choroid plexus [1, 2]. Magnetic resonance imaging (MRI) is the preferred imaging modality for assessing the central nervous system because of its high soft-tissue contrast and because it does not use ionizing radiation. However, unequivocal identification of intracranial calcifications with MRI is challenging. On conventional T_1_-, T_2_-weighted spin-echo MR images calcifications appear with various signal intensities, which makes differentiation of calcified tissue from other sources difficult [3, 4].

The development of susceptibility-weighted imaging (SWI) and phase imaging, two gradients recalled echo (GRE) imaging post-processing techniques, has enabled to discriminate lesions based on their magnetic susceptibility [5–7]. Blood degradation products (like hemosiderin) are more paramagnetic compared to the brain parenchyma, while calcifications exhibit more diamagnetic susceptibilities [5, 8]. Thus, SWI and phase imaging allow us to differentiate between calcifications and other causes of susceptibility differences [4, 9].

Higher incidences of brain calcifications in the hippocampus and basal ganglia are found in patients with neurodegenerative diseases such as Alzheimer’s disease (AD), cerebral amyloid angiopathy, frontotemporal dementia, Parkinson’s disease and Down syndrome [10–12]. In patients with diffuse neurofibrillary tangles with calcification, pallidal calcification is the most prominent feature and is accompanied by frontotemporal atrophy, neurofibrillary tangle deposition throughout the neocortex, without the occurrence of β-amyloid plaques [13]. Despite the high prevalence of intracranial calcification in neurodegenerative disorders, their etiology is yet poorly understood.

The discovery of tau mutations has facilitated the generation of several mouse models of human tauopathy e.g. P301S and P301L lines (transgenic for a human 4 repeat tau isoform). These models have become important tools to study the mechanisms of abnormal tau aggregation and deposition in frontotemporal dementia (4 repeat tau) and AD (3 and 4 repeat tau) [14, 15]. In the P301L mouse models, tau deposits begin forming before 3 months-of-age in neurons in the entorhinal cortex, hippocampus, and later in the cortex, and amygdala [16–19]. Neuroinflammation and impaired memory functions in the hippocampus- and amygdala-dependent tasks manifest at a later stage [20, 21]. Brain atrophy and white matter changes, indicating neurodegeneration, were reported to occur at around 9 months-of-age [22]. However, unlike in human patients with tauopathies, brain calcifications have not been reported in transgenic mouse models of human tauopathies.

Here, we describe the occurrence of intracranial calcifications as a new phenotype in transgenic P301L (Thy1.2) mice overexpressing four repeat tau, a model of human tauopathy. All mice were first investigated with in vivo MRI, and head samples were obtained for ex vivo MRI and micro-CT (μCT). We verified the nature of the lesions by histochemistry and immunohistochemistry.

## Materials and methods

### Animals

All experiments were performed in accordance with the Swiss Federal Act on Animal Protection and approved by the Cantonal Veterinary Office Zurich (permit number: ZH082/18). Homozygous mice, transgenic for a human four repeat isoform with the P301L under Thy1.2 promoter (C57B6.Dg background) [16, 17], and non-transgenic littermates were used (see groups in Supplementary Table 1). Animals were housed in individually ventilated cages inside a temperature-controlled room, under a 12-h dark/light cycle. Pelleted food and water was provided ad-libitum.

### Magnetic resonance imaging

In vivo MRI was completed on a horizontal Bruker Biospec 7 T (Bruker Biospin GmbH, Ettlingen, Germany) small animal MR system equipped with an actively shielded gradient set of 760 mT/m and 80 μs rise time and a volume transmit coil of 72 inner diameters and an actively decoupled 2 × 2 surface array covering 20 mm × 24 mm for signal reception (Bruker BioSpin AG), operated by a Paravision 6.0.1 software platform (Bruker Biospin GmbH, Ettlingen, Germany). Mice were anesthetized with an initial dose of 4% isoflurane (Abbott, Cham, Switzerland) in oxygen/air (200:800 ml/min) and maintained at 1.5% isoflurane in oxygen/air (100:400 ml/min). Body temperature was monitored with a rectal temperature probe (MLT 415, AD Instruments, Spechbach, Germany) and kept at 36.5 ± 0.5 °C on a water-heated holder (Bruker BioSpin AG, Fällanden, Switzerland). A two-dimensional flow compensated gradient-recalled echo (FLASH) sequence was applied with the following parameters: field-of-view = 20 × 20 mm; image size = 256 × 256 mm, slice thickness = 0.8 mm, resulting in a resolution of 78 × 78 µm, number of slices = 20. One echo with an echo time = 18 ms; repetition time = 698 ms; flip angle = 30°; and the number of averages = 30 within an acquisition scan time of 1 h 29 min 2 s was recorded. To reduce field inhomogeneities we first shimmed globally, followed by a localized shim over the brain with the Mapshim protocol, which uses a field-map (default protocol settings), using the first and second-order shims.

After in vivo MRI, mice were intracardially perfused under ketamine/xylazine/acepromazine maleate anesthesia (75/10/2 mg/kg body weight, i.p. bolus injection) with 0.1 M phosphate-buffered saline (PBS, pH 7.4, Sigma Aldrich, Switzerland) and decapitated. The mouse heads were post-fixed in 4% paraformaldehyde (Sigma Aldrich, Switzerland) in 0.1 M PBS (pH 7.4) for 6 days and stored in 0.1 M PBS (pH 7.4) at 4 °C afterward.

Ex vivo MRI was done on a horizontal Bruker Biospec 9.4 T (Bruker Biospin GmbH) small animal MR system. The system was equipped with a cryogenic 2 × 2 radiofrequency surface coil probe (Bruker BioSpin AG). The heads were then placed in a 15 ml centrifuge tube filled with perfluoropolyether (Fomblin Y, LVAC 16/6, average molecular weight 2700, Sigma-Aldrich, U.S.A.). Since it is a proto-free compound it renders a dark background on MRI. Samples were measured at room temperature. A 3D gradient-recalled echo SWI sequence was recorded with the following parameters: field-of-view = 15 × 12 × 15 mm; image size = 248 × 200 × 36 mm, resulting in a spatial resolution of 60 × 60 × 417 µm. One echo with an echo time = 12 ms; repetition time = 250 ms; flip angle = 15°; number of averages = 4 within an acquisition scan time of 1 h 59 min 24 s was recorded. A global and MAPSHIM protocol with a field map (default setting) was used for shimming.

### Data post-processing and analysis

The datasets generated and/or analyzed during the current study are available in the repository (https://doi.org/10.5281/zenodo.3518986). SW and phase images were computed using the SWI processing module in ParaVision 6.0.1 (Bruker, Ettlingen, Germany) with the Gauss broadening = 1 mm and a mask weighting = 4. All SW images were compared with their phase image counterparts to ensure that the signals were diamagnetic (i.e. presence of calcifications). The numbers of suspected calcified spots in a panel of an anatomical region (hippocampus, thalamus, caudate nucleus, midbrain, and cortex etc.) were quantified using the ex vivo datasets acquired at 9.4 T. The Allen mouse brain atlas was used for anatomical reference [23].

### µCT

Mouse heads were scanned in 0.1 M PBS (pH 7.4) in a μCT 40 (Scanco Medical AG, Brüttisellen, Switzerland) operated at 45 kVp, 177 µA intensity, an integration of 200 ms and twofold frame averaging. From 1000 projection images, 3D datasets with isotropic voxels of 8 µm were reconstructed and converted to DICOM. DICOM files were exported and analyzed using ITK SNAP [24].

### Histochemistry and immunohistochemistry

Additional mice that did not undergo MRI examination were intracardially perfused with PBS and paraformaldehyde as described above. Heads/brains were postfixed for 6 h and were stored in PBS until further use. Fixed mouse brains were cut sagittally into two hemispheres. One hemisphere was cut into 60 μm coronal sections using a vibratome (Leica VT1000S, Germany) for fluorescence immunohistochemistry. The protocol used for fluorescence immunohistochemistry was described previously [9, 22]. Primary antibodies used for immunofluorescence staining are listed in Supplementary Table 2. AT8 and AT-100 antibodies bind to different tau isoforms. While AT-8 binds to pSer202/pThr205 epitope and detects sarkosyl-insoluble tau and soluble hyperphosphorylated tau [25], AT-100 binds to pThr212/pSer214 epitope, recognizes sarkosyl-insoluble, but not soluble tau [26]. Fluorescently labelled secondary antibodies: donkey anti-goat Cy3, donkey anti-rat DyLight 649, donkey anti-mouse Alexa 488, and donkey anti-rabbit Alexa 488 (Jackson Immunoresearch, UK) were used. Brain sections were pre-treated with the mouse-on-mouse kit (Vector Laboratories, USA) to quench the endogenous IgG and subsequently incubated with primary and secondary antibodies. Immunohistochemistry stainings were imaged using a confocal microscope (Leica SP5; 40 × numerical aperture: 1.25; 63 × numerical aperture: 1.4). Images were analyzed using the image-processing software Imaris 8.4.1 (Bitplane, USA) and Illustrator CS 6 (Adobe, USA).

For histochemistry and non-fluorescent immunostaining, the other brain hemisphere was embedded in paraffin following routine procedures and cut into 2 μm thick sections. Sections were stained using Hematoxylin & Eosin (HE), Alcian blue, Periodic acid–Schiff or Prussian blue using a standard protocol. The sections were deparaffinized and rehydrated before immunostaining. For glial fibril acidic protein (GFAP) staining, sections were incubated with rabbit anti-GFAP (DakoCytomation A/S, Denmark, #20334), followed by incubation with HRP-conjugated goat anti-rabbit (Jackson Immunoresearch, USA). For ionized calcium-binding adaptor molecule 1 (Iba1) staining, antigen retrieval was performed using hot citrate buffer (0.01 M; pH 6), followed by incubation with rabbit anti-Iba1 (WAKO, Japan; 1:2500) and subsequently a biotinylated secondary antibody (Vector laboratories, USA). Visualization ensued after using the ABC complex solution (Vector laboratories, USA), 3,3′-Diaminobenzidine (Sigma-Aldrich, Switzerland), and hydrogen peroxide (Sigma-Aldrich, Switzerland). Counterstaining was performed using Hematoxylin. Stained paraffin sections were scanned using a NanoZoomer HT (Hamamatsu Photonics, Japan) using a 40 × objective. Images were analyzed using Digital Image Hub software (SlidePath) and Adobe Illustrator CS6 (Adobe, USA). HE stained sagittal mouse brain slices were imaged at 20 × magnification using Pannoramic 250 (3D HISTECH, Hungary). The images were analyzed using CaseViewer (3D HISTECH, Hungary) and ImageJ (NIH, USA).

### Statistics

Statistical analysis was performed using GraphPad Prism 7.0 (GraphPad Software, USA). D’Agostino and Pearson normality test was used for assessing the normal distribution of the data. One-way ANOVA with Tukey’s post-hoc analysis was used for group comparison. The difference between groups was considered significant (*) at *p* value < 0.05. All error bars in the figures are expressed as standard deviation.

## Results

### In vivo SWI and phase imaging at 7 T detect the presence of diamagnetic lesions in brain of P301L mice

**Fig. 1:**
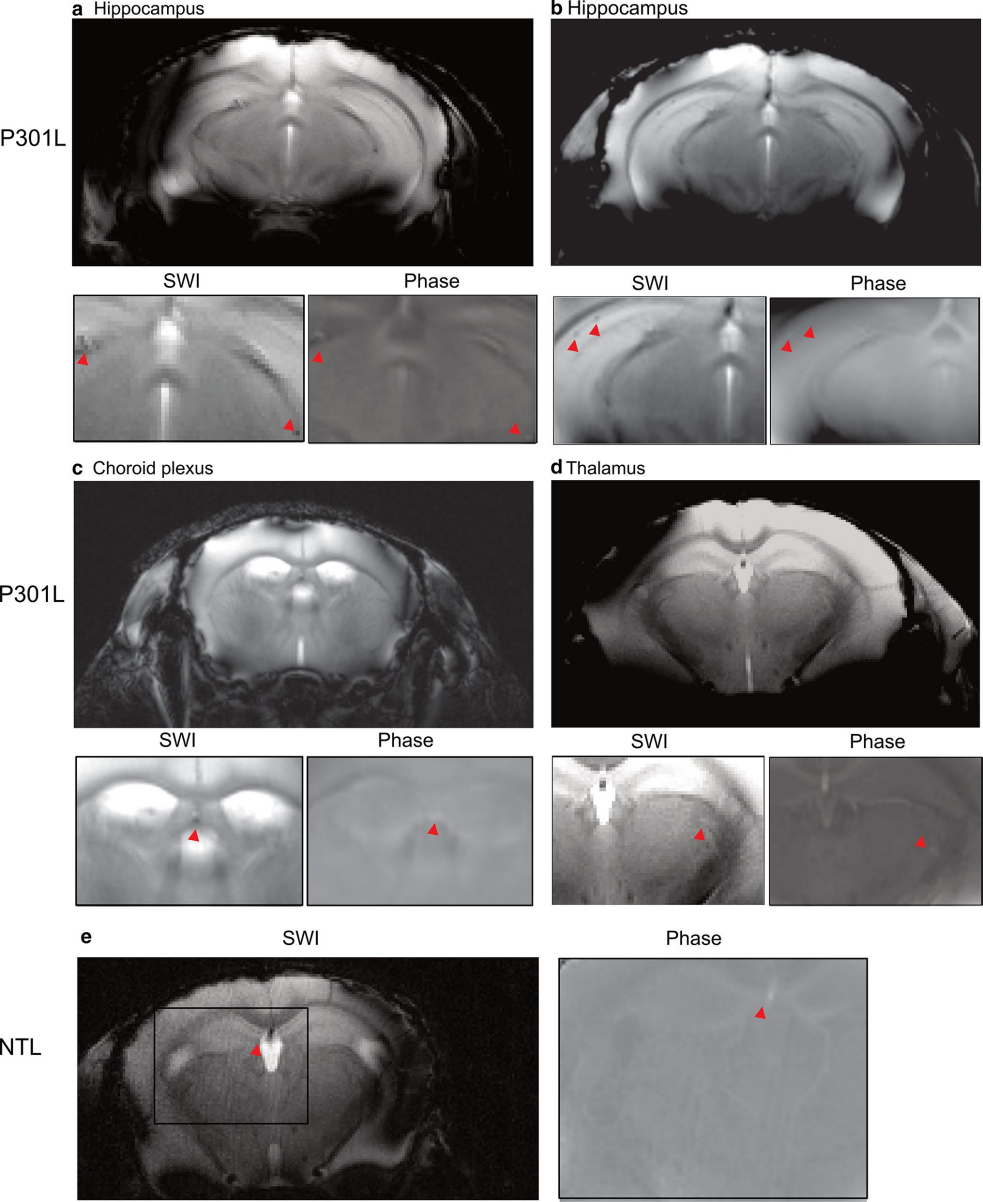
In vivo SWI and phase imaging reveals diamagnetic lesions in vivo in P301L mouse brain. Representative in vivo SW and phase image at 7 T showing hypointensities/positive phase shifts (red arrowheads) in the **a, b** hippocampus in two 18 month-old P301L mice, **c** choroid plexus (of the third ventricle) and **d** thalamus in one 18 month-old P301L mouse, **e** hypointensities/positive phase shifts were only seen in the choroid plexus in one representative 11 month-old non-transgenic littermates (NTL)

First, we collected in vivo gradient recalled echo data of P301L mice and non-transgenic littermates for phase and SWI post-processing (Fig. 1a-d). We observed hypointensities in the SW images in P301L mice. Corresponding phase images showed positive phase shifts (hyperintensities) in corresponding locations, indicating the diamagnetic nature of lesions. The magnetic susceptibility and appearance of the lesions indicated the presence of calcified deposits [9, 27]. Hypointensities/positive phase shifts in P301L mice were detected in all age groups, prominently in the hippocampus, but also in the thalamus, caudate nucleus, choroid plexus and midbrain (Fig. 1, red arrows). In comparison, SW and phase images of aged non-transgenic littermates hypointensities/positive phase shifts were only observed in the choroid plexus (Fig. 1e, red arrow).

### Ex vivo SWI and phase imaging detect higher load of diamagnetic lesions

**Fig. 2:**
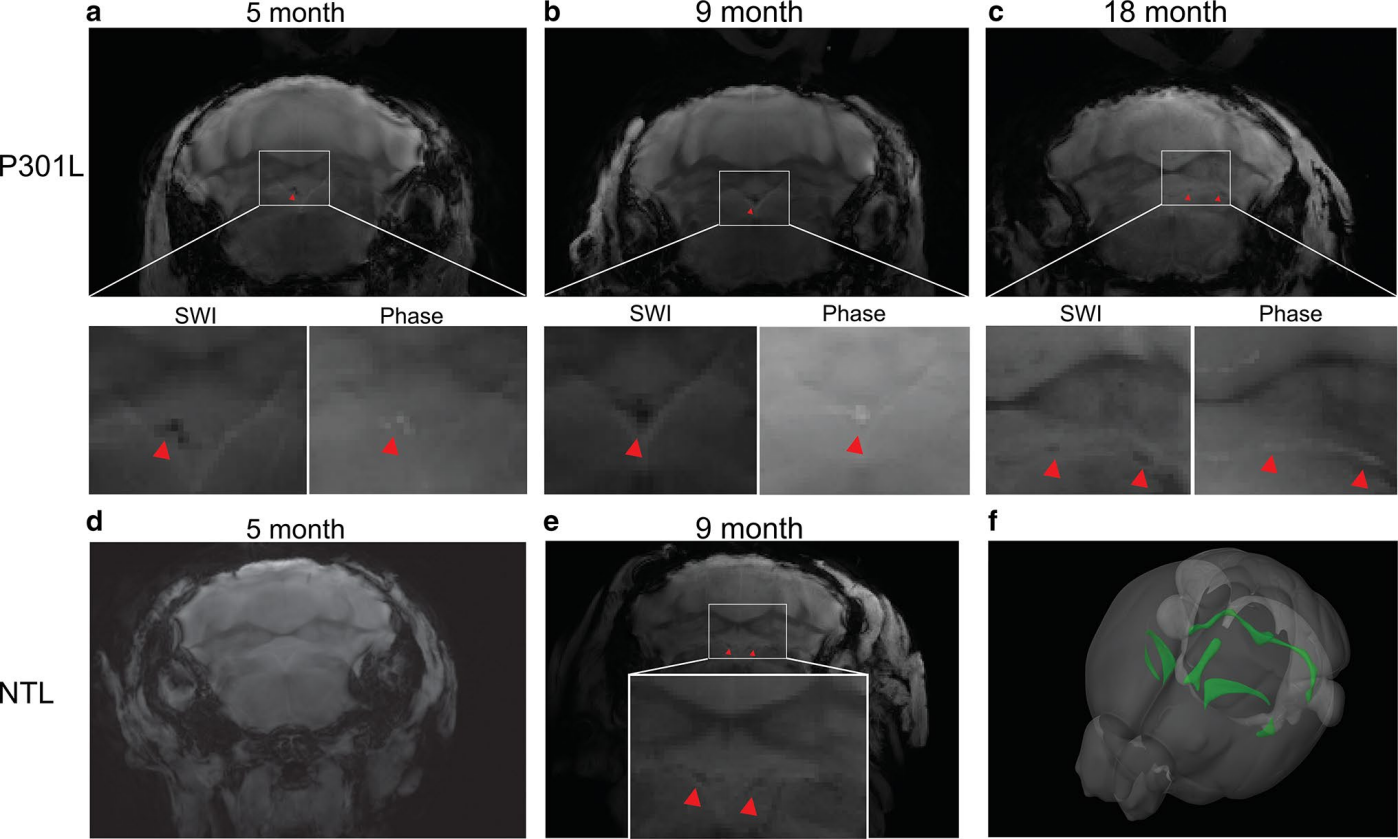
Comparison of ex vivo imaging findings in the choroid plexus of P301L and non-transgenic mice. **a**–**c** Representative ex vivo SWI at 9.4 T and corresponding phase image showing hyperintensities in the fourth ventricle choroid plexus of 5, 9 and 18 month-old P301L mouse; **d, e** representative ex vivo SW images at 9.4 T in the fourth ventricle choroid plexus of 5 month-old and 9 month-old non-transgenic littermates (NTL); **f** 3D mouse brain atlas from Allen Institute with choroid plexus highlighted in green [23]. Intracranial calcifications are marked by a red arrowheads

**Fig. 3:**
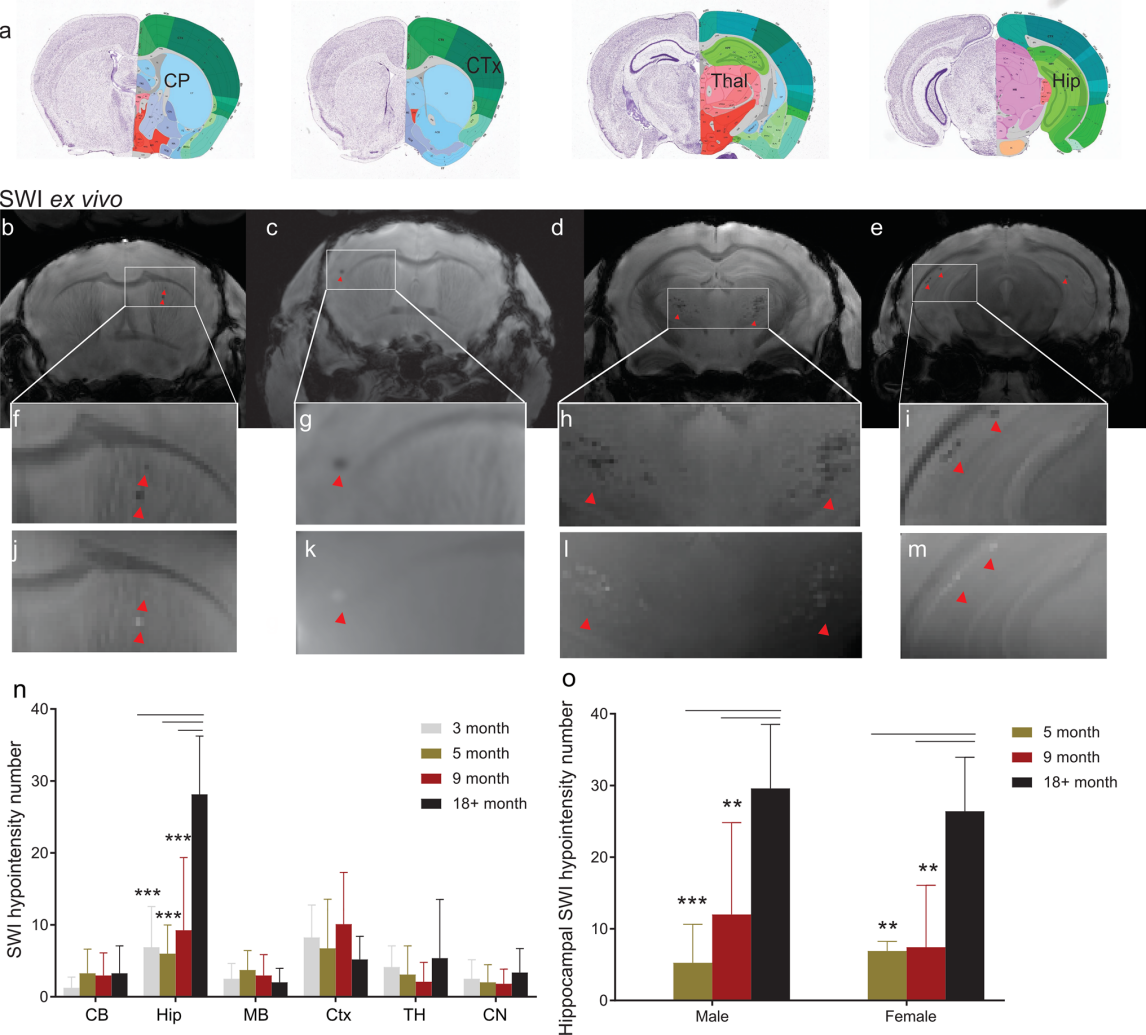
SWI and phase imaging reveals diamagnetic lesions ex vivo brain of P301L mice. **a** Corresponding anatomical sections from Allen Institute mouse brain atlas (slices 46, 56, 76, 89) [23] for SW images shown in **b–e**; **b–i**: representative ex vivo susceptibility-weighted (SW) and **j–m** phase image at 9.4 T showing (**b, f, j**) hypointensities/positive phase shifts in the 5 month-old P301L mouse; (**c, g, k**) in the cortex of a 9 month-old P301L mouse; **d, h, l** in the thalamus of a 18 month-old P301L mouse; **e, i, m** in the hippocampus of a 18 month-old P301L mouse (red arrowheads); **n** quantification of regional distribution of SW hypointensities. 18 + month (*n* = 11) compared to 3 month (*n* = 4, *p* < 0.0001), 5 month (*n* = 11, *p* < 0.0001), 9 month (*n* = 10, *p* < 0.0001); **o** number of SWI hypointensities in male and female P301L mice of 5, 9 and 18 + month-old animals **p < 0.01, ***p < 0.001 two-way ANOVA with Tukey’s post hoc analysis

Ex vivo studies have the advantage in that they allow for longer acquisition times, thus achieving higher spatial resolution. In addition, embedding heads in perfluoropolyether fills air cavities (e.g. the ear canals) that give rise to susceptibility artefacts in nearby brain regions (Fig. 1). In addition, we took advantage of using 9.4 T and a cryogenic radiofrequency coil to achieve higher signal-to-noise ratios. Hypointensities in the SW images in P301L mice on reconstructed data from ex vivo MRI at 9.4 T (Figs. 2, 3) correspond well in general to that from in vivo MRI at 7 T (Fig. 1). Boundaries of lesions were sharper in the 9.4 T compared to 7 T images, however, a one-to-one comparison was not made due to different parameters in the acquisition protocol. In both P301L mice and non-transgenic littermates, lesions were observed in the choroid plexus (e.g. positive phase shifts in the fourth ventricle, which appeared as a cohesive structure) (Fig. 2a-d). In addition, hyperintensities/positive phase shifts on SW/phase images appeared in the P301L mice as single (Fig. 3f, g, i, j, k, m), or clustered structures (Fig. 3h, i, l, m).

We next quantified a load of observed cerebral SWI hyperintensities in P301L mice at different ages. Application of the D’Agostino and Pearson normality test yielded *p* = 0.0648, indicating passing the normality test. The number of SW/phase diamagnetic deposits increased with age in the hippocampus, but not in the other brain structures (18 months-old vs 3 months-old, *p* < 0.0001; vs 5 months-old, *p* < 0.0001; and vs 9 months-old, *p* < 0.0001, Fig. 3n). No statistically significant difference was observed between male and female mice within age groups (Fig. 3o).

### µCT detects hyperdense lesions in the brain of P301L mice

**Fig. 4:**
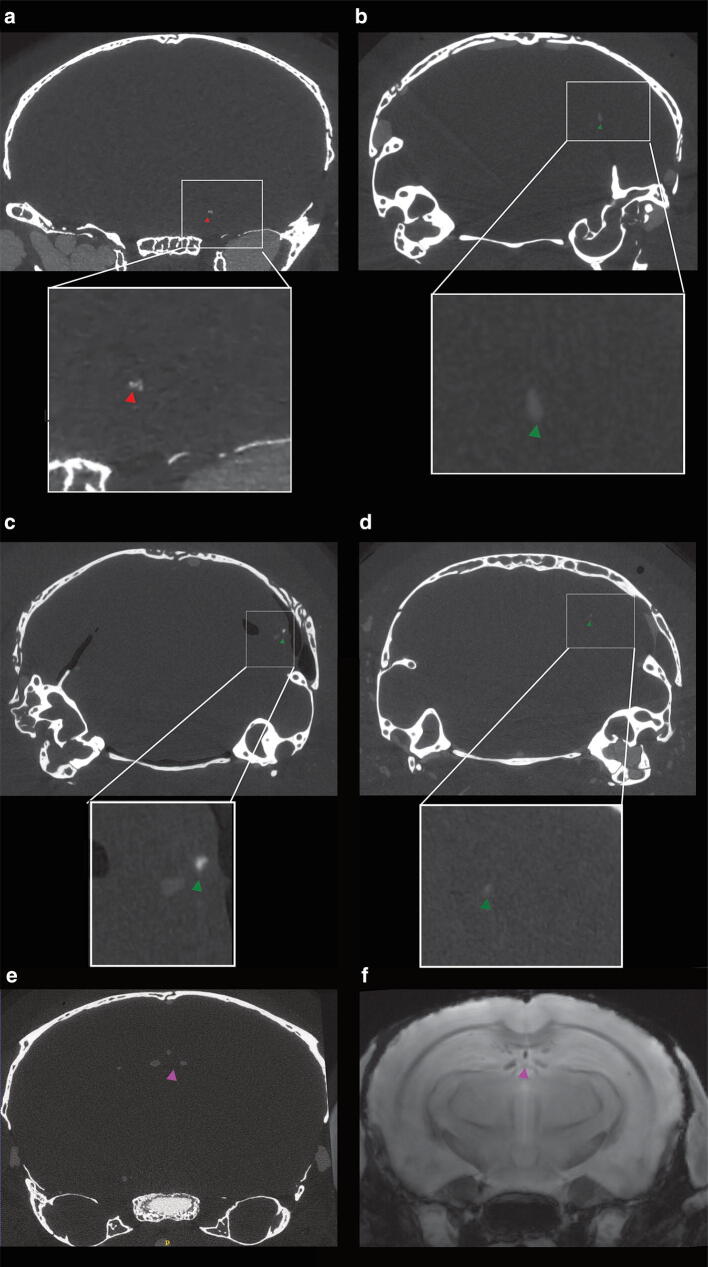
Hyperdense lesion detected on μCT images of the brains of P301L mice. Representative hyperdense lesions in 5 different P301L mice **a** hippocampus of one 18 month-old P301L mouse (red arrowhead), **b, c** cerebellum of two 5 month-old p301L mice, **d** cerebellum of one 9 month-old P301L mouse (green arrowhead), **e, f** corresponding ex vivo microCT and MRI in the third ventricle of one 18 month-old P301L mouse (purple arrowhead)

To further assess cerebral lesions in the brain of P301L mice, ex vivo μCT scans were performed on the same brain samples. Hyperdense lesions were observed in 5, 9- and 18 + months-old P301L mice. The higher X-ray density in these areas indicates the presence of material of a higher atomic number than soft tissue [28]. However, fewer lesions were observed using μCT compared to MRI. Hyperdense lesions were seen in regions that correspond to the hippocampus (Fig. 4a), cerebellum (Fig. 4b-d), and the choroid plexus of the third ventricle (Fig. 4e, f) in P301L mice. This adds additional evidence for the presence of intracranial calcification in P301L mice.

### Vascular bone protein-containing nodules in the thalamus of P301L mice

**Fig. 5:**
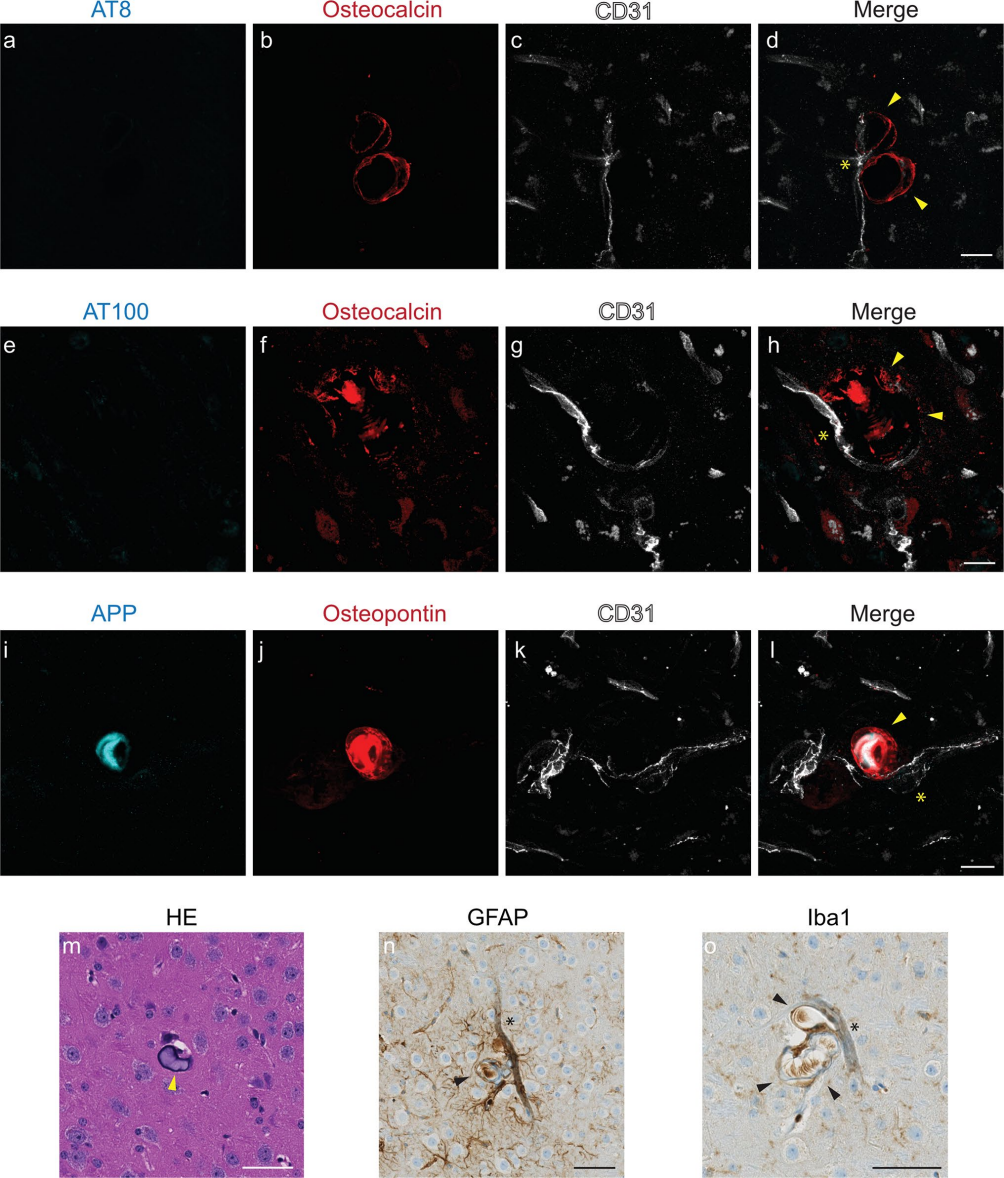
Characterization of vessel-associated nodules in the thalamic region of 18 month-old P301L mice** (a-l**): immunohistochemical identification of the lesions. Osteocalcin-positive (red) nodules (**b, f**) are associated with vessels (**c, g,** CD31; white) and do not stain with antibodies recognizing different phosphorylated residues of tau, AT8 (**a)** and AT100 (**e**); thalamic nodules are positive for APP (**i,** cyan) and osteopontin (**j**, red); **m** HE stain of thalamic nodules; **n, o** thalamic nodules elicit glial reactivity. Activated astrocytes (**n**) and microglia (**o**) surround vascular nodules. Vessels are visualized using CD31 (**c, g, k**, white) and vessel adjacent to bone protein containing nodule is marked using an asterix (**d, h, l**). Arrowheads (**d, h, l**) mark thalamic nodules. Scale bars: 15 µm (**a–l**) and 50 µm (**m–o**)

As a next step, we used histochemistry and immunohistochemistry to investigate the nature of the imaging lesions in different brain regions of P301L mice. To this end, we used immunohistochemical staining with antibodies against bone proteins deposited in brain calcifications [9, 29]. In the thalamus, osteocalcin- and osteopontin-positive nodules were associated with blood vessels, in the absence of phosphorylated-tau (AT8 and AT100) staining (Fig. 5a–h). Interestingly, similar to previous reports [30], we detect an amyloid-precursor protein (APP), a marker for damaged neurons [31], deposition in vascular nodules containing bone proteins (Fig. 5i-l). These nodules were visualized using HE staining, a standard histological stain, indicating the basophilic nature of these structures (Fig. 5m). Iba-1 reactivity was seen in the brains of aged P301L mice. GFAP reactivity was seen in cortical areas. In addition, vascular calcification in the thalamus elicits a strong focal glial reactivity (Fig. 5n, o).

We performed histology on sagittal brain sections of P301L mouse and non-transgenic littermate brains to verify the presence of other neuropathology in the brain of P301L mice. Alcian blue, Periodic acid–Schiff, and HE staining showed no pathological abnormalities or infarction in the mouse brain P301L mice at 18 month-of-age (SFig. 1). Furthermore, Prussian blue staining demonstrated that the imaging pattern in the P301L mouse brains were not cerebral microbleeds (SFigs. 1c, d).

### Vascular and intracellular bone protein-containing nodules in the hippocampus

**Fig. 6:**
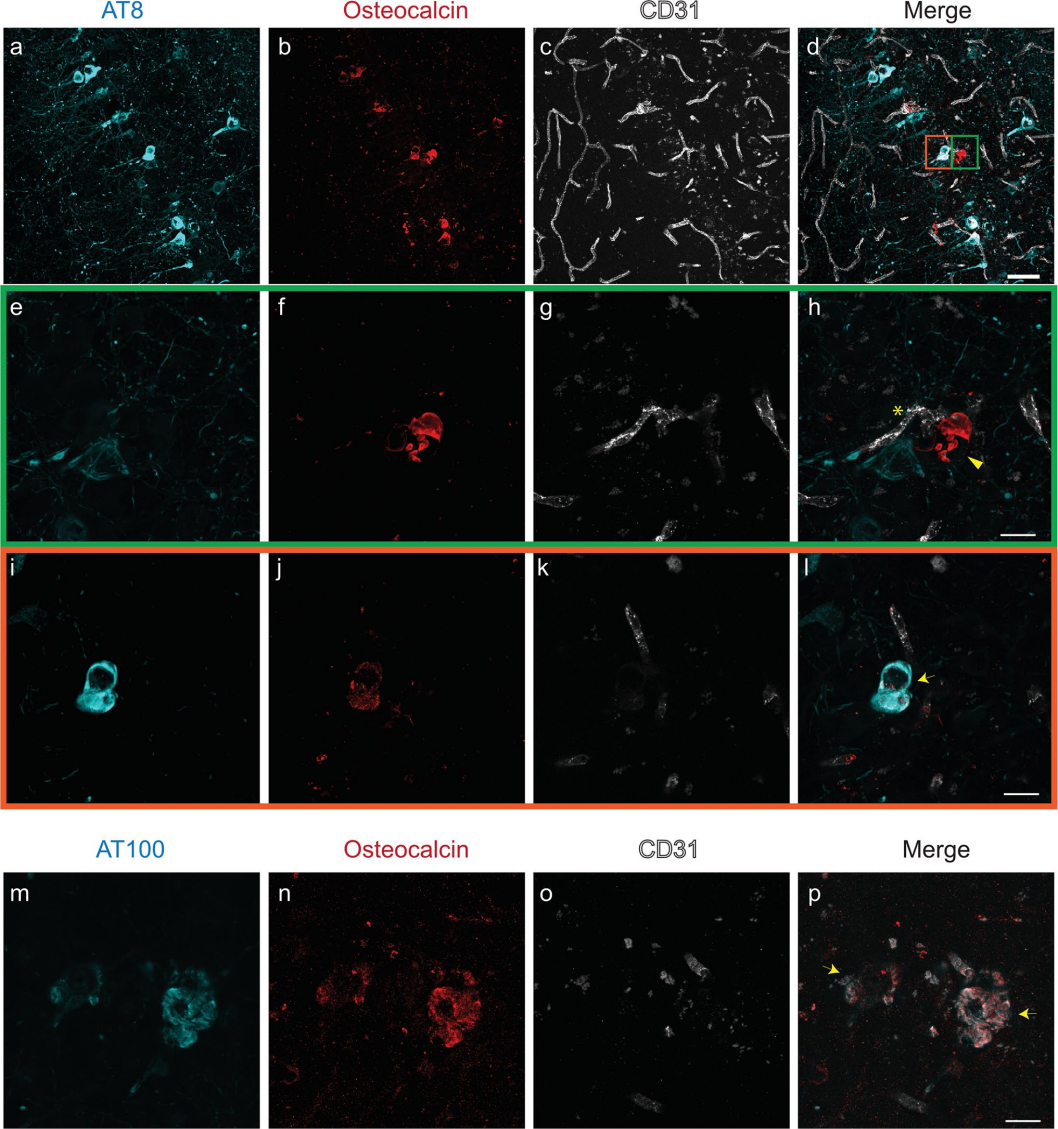
Characterization of hippocampal inclusions in 18 month-old P301L mice (**a–p**). Osteocalcin (**b, f, j**, red) staining in the hippocampus. (**e–h**, green inset) Extracellular osteocalcin-positive (**f**, red) nodule is vessel-associated (**g**, CD31; white), but did not stain with the phosphorylated-tau AT8 (**e**, cyan) antibody. (**i–l**, orange inset) Intracellular co-localization of phosphorylated-tau AT8 (**i**, cyan) and osteocalcin (**j**, red) in a hippocampal neuron; (**m–p**) Co-localization of osteocalcin (**n**, red) positivity with phosphorylated-tau AT100 (**m**, cyan) in the hippocampus of P301L mice. Vessels are visualized using CD31 (**c, g, k, o**, white) and the vessel adjacent to nodule is marked using an asterix (**h**). Arrowheads (**h**) mark extracellular nodules and arrows (**l, p**) mark intracellular co-localization of phosphorylated-tau stains and osteocalcin. Scale bars: 50 µm (**a–d**) and 15 µm (**e–p**)

The hippocampus of P301L mice was found to be a prominent site of imaging pattern indicative of tissue calcification (Fig. 3). Immunohistochemistry using antibodies against bone proteins confirmed their presence in deposits (Fig. 6a–p), which were vascular (Fig. 6e–h) and parenchymal (Fig. 6i–l). In contrast to the thalamus, we observed in the hippocampus of P301L mice intracellular staining of osteocalcin which was co-localizing with phosphorylated-tau detected using antibodies AT8 (sarkosyl-insoluble tau and soluble hyperphosphorylated tau) and AT100 (sarkosyl-insoluble tau) (Fig. 6i–l).

## Discussion

In this study, we observed intracranial calcifications on SWI and phase images in the brains of P301L mice. Immunohistochemistry, which revealed that bone protein-containing lesions were either vessel-associated or intracellular and co-localizing with phosphorylated-tau.

In the present work, we used MRI to detect calcifications in P301L mice and compared detection with μCT. SWI and phase imaging allows detecting tissue calcifications based on the magnetic susceptibility of the lesions [5]. In our study, all hypointense inclusions on SW images of the P301L mouse brain were diamagnetic, indicating the presence of intracranial calcifications (Figs. 1–3). Immunohistochemistry revealed that lesions are positive for bone proteins, thus indicating that the characteristic imaging pattern corresponds to markers of calcified tissue (Figs. 5, 6). Blood degradation products such as hemosiderin are paramagnetic and would induce opposite signal shifts [8]. Negativity for Prussian blue staining indicated the absence of cerebral microbleeds and other iron deposition in the brain of P301L mice (SFigs. 1c, d).

In non-transgenic littermates, imaging abnormalities were found confined to the choroid plexus. While this has been reported to occur in aged individuals [2, 32], it has so far not been reported as a phenotype in aged mice. In P301L mice we observed high inter-individual variations in hypointensities on SW images in all anatomical regions (Fig. 2g), similar to previous studies in patients with familial brain calcification and mouse models thereof [9, 27].

Studies using clinical brain imaging data suggest that the diagnostic accuracy of SWI outperformed conventional MRI and CT in detecting brain calcifications [3, 4], although the diagnostic accuracy of different methods might depend on disease indication, exact MRI parameters and field strengths used. In our study, we observed much fewer calcifications on μCT images compared to MRI (Fig. 4a-d). We used a μCT system optimized for achieving high spatial resolution (8 μm vs 78 μm and 60 μm in MRI, respectively). For MRI, we worked at 7 T and 9.4 T, which provides a large signal-to-noise ratio and optimal detection of susceptibility differences (and thus phase and SWI contrast) [7]. The differences in the detectability of intracranial calcifications may be due to the fact that the calcifications are small and less dense than bone structures e.g. the skull bone and are thus more difficult to detect with μCT. In both SWI and phase imaging nonlocal field perturbations of the diamagnetic inclusions lead to blooming effects [7], which might facilitate the detection of calcifications. However, this also implies that, compared to CT, SWI, and phase image do not allow estimating the true size of calcifications. In addition, macroscopic field inhomogeneities increase as well with increasing field strengths, leading to geometric distortions and intravoxel dephasing [7]. In our study, however, calcifications were not observed in regions that are particularly prone to susceptibility artifacts (i.e. air tissue interfaces). One limitation of GRE phase imaging and SWI is the nonlocal nature of phase effects. The signal phase will also be affected in areas that extend beyond the actual size of the dia- or paramagnetic inclusion. Moreover, phase values depend on the geometry of the lesion and its relative orientation to the main magnetic field [33]. These limitations may be overcome by converting GRE phase images into susceptibility maps. Quantitative susceptibility maps (QSM) represent an intrinsic tissue property and are independent of data acquisition parameters and should reflect the actual spatial extent of lesions. The feasibility of QSM for the depiction of the cerebral lesion has been shown in a number of studies [8, 34–36]. However, in contrast to QSM post-processing, procedures for SWI are established on clinical scanners and can be readily applied for the detection of calcifications and other brain lesions.

The etiology of most forms of brain calcifications are unknown, but they may occur under several pathophysiological conditions, including inflammation, metabolic, infectious and genetic syndromes, and after exposure to toxins or radiotherapy [37]. Loss-of-function mutations in genes involved in familial forms of diseases presenting with brain calcification have been associated with disturbance in phosphate homeostasis and the dysfunction of the blood–brain barrier [38]. Moreover, studies using hypomorphs of platelet-derived growth factor subunit B suggested a connection between blood–brain barrier impairment and brain calcification [27]. Interestingly, blood–brain barrier breakdown has been reported in P301L tauopathy mice, with erythrocyte and leukocyte infiltration occurring before the accumulation of hyperphosphorylated tau [39]. Thus, it would be of interest for future studies to investigate if blood–brain barrier impairment plays a role in the manifestation of brain calcification in this setting. In addition, while glial reactivity was generally found in the brain of aged P301L mice, we observed strong glial reactivity towards calcified nodules in the thalamus of the P301L mice (Fig. 5n, o), which might implicate also a role for reactive astrocytes and microglia in the progression of the pathology [30].

Intracranial calcification may also be linked to tauopathy. Studies demonstrated hippocampal calcification in the brain of patients with AD and frontotemporal dementia using CT and histopathology [10, 40]. This is in line with our study where hypointensities were most abundant in the hippocampus, increasing in an age-related manner (Fig. 3j). Furthermore, we found co-localization of tau (AT8, AT100) with osteocalcin in hippocampal neurons, the earliest brain region affected in AD and frontotemporal dementia (Fig. 6d, l). In P301L mice, tau accumulation is prominent in the hippocampus, amygdala, and cortical regions but sparse in the brain stem, thalamus, and caudate nucleus [16–19]. The tau distribution was not matched regionally with the hypointensity pattern seen on MR imaging at the whole brain level (Figs. 1, 2, 3). The susceptibility to developing calcification may thus depend on a number of other factors in addition to human tau overexpression, such as the biochemical, and genetic properties of the tissue. The individual and combined contribution of these factors to the formation of brain calcification needs further research [41]. Given the non-invasive nature of the MRI, longitudinal studies would be informative with respect to individual changes in calcification load and can be combined with other read-outs (e.g. perfusion measurement, elastography etc.).

We observed APP and osteopontin accumulation in the thalamic nodules in P301L mice. APP is a marker for damaged neurons [31], and osteopontin is a bone protein upregulated in the damaged brain [42]. Both APP [31], and osteopontin [29], are reported in the extracellular matrix of calcified tissue. The presence of APP could be an indication of damaged neurons in the area. Here, we observed that they accumulate in the thalamic calcifications in P301L mice similar to other reports about brain calcifications, further supporting that these nodules are indeed calcified. As the antibody for APP binds to amino acids 85–99 of the C99 fragment of APP, it could detect both amyloid-beta deposits and APP C-terminal fragment C99 located in dystrophic neurites.

Once manifested, brain calcification can affect brain function by evoking expression of neurotoxic astrocyte markers, interfering with neuronal circuitry and/or glucose metabolism [3, 9]. However, the functional contribution of brain calcification to neuropsychiatric symptoms in various brain disorders is still debated [37, 43]. Patients with primary familial brain calcification often present with impaired movement (Parkinsonism and dystonia), but also cognitive impairment and psychiatric manifestations, including schizophrenia-like symptoms, mood disorders, or obsessive–compulsive disorder [44]. Patients with diffuse neurofibrillary tangles with calcification show early but progressive memory and verbal disturbances, followed by psychiatric symptoms. While the P301L strain shows impaired memory functions in hippocampus- and amygdala-dependent tasks [20, 21], future studies need to disentangle the potential contributions of tauopathy-related brain calcification to functional deficits.

### Limitations

We did not systematically compare the sensitivity of detecting intracranial calcifications at different field strengths. It is expected that the use of higher field strengths (i.e. 9.4 T compared to 7 T) improves the detectability of lesions because the phase scales linearly with the field, and due to an increase in signal-to-noise ratio [7]. However, we have also used different radiofrequency coils and protocols, which hampers to make a direct comparison. For in vivo MRI we used a 7 T scanner with a surface array coil and a short imaging protocol to minimize exposure of the animal to anesthesia. For ex vivo MRI we used at 9.4 T with a cryogenic surface coil to use achieve a high signal-to-noise ratio. While 7 T constitutes a field strength that is available in clinical settings, most clinical scanners work at lower field strength and we thus expect that (small) calcifications are difficult to detect, though the detection of calcifications with MRI has been reported [3, 4]. Assessment of the morphology of individual calcifications was difficult in ex vivo SW/phase images given the chosen slice thickness. Using isotropic voxels with a high spatial resolution would improve the assessment of such lesions.

SWI and phase imaging has been found to be more sensitive to detect intracranial calcifications in the P301L model than μCT, which is in line with previous clinical reports [3, 4]. This may be due to the specific composition of calcifications in this pathology. While we detected protein-positive deposits with immunohistochemistry, we do not know to which degree this resulted in the deposition in calcium carbonate and calcium phosphate and their respective density of distribution, which is relevant for the detection with SWI, phase, and CT. Thus, further studies should aim to elucidate the relationship between the built-up of calcifications i.e. changes in chemical composition and density and its detectability with MRI and CT.

## Conclusions

We described intracranial calcifications as a new brain phenotype of P301L mice. SWI and phase imaging can sensitively detect these types of calcifications. The study further suggests a potential link between tau deposition and tissue calcification and the P301L mouse strain may be a suitable model to study the pathogenesis and pathophysiology of brain calcifications in frontotemporal dementia and AD, and other tauopathies.

## Electronic supplementary material

Below is the link to the electronic supplementary material.

Supplementary file1 (DOCX 15 kb)

Supplementary file2 (TIF 28324 kb)
