# Supplementary material for: SWI and phase imaging reveal intracranial calcifications in the P301L mouse model of human tauopathy
Source: MAGMA. 2020 May 28;33(6):769–81. doi: 10.1007/s10334-020-00855-3 (PMC7669813; doi:10.1007/s10334-020-00855-3)
Supplement: Supplementary file 1 — Supplementary file1 (DOCX 15 kb) [file 10334_2020_855_MOESM1_ESM.docx]

**Supplementary table 1 Summary of transgenic and non-transgenic mice used in the study**

|  | Imaging | | Immunohistochemistry | |
| --- | --- | --- | --- | --- |
|  | Homozygote (P301L) | Non-transgenic | Homozygote (P301L) | Non-transgenic |
| 3 month | 4M |  |  |  |
| 5 month | 5F/6M | 4F/1M | 1M/1F | 1F |
| 9 month | 6F/4M | 4F/1M | 2M/2F | 1M |
| 18+ month | 5F/6M |  | 1M/2F |  |

M: male; F: female; Homozygote and non-transgenic mice have the C57B6 background.

**Supplementary table 2. List of primary antibodies used for immunohistochemistry**

| **Antibody** | **Company** | **Cat. No.** | **Dilution** |
| --- | --- | --- | --- |
| Goat anti-Osteocalcin | Alfa Aesar | J65216 | 1:500 |
| Rabbit anti-APP | ThermoFischerScientific | PA5-19923 | 1:200 |
| Mouse anti-AT100 | ThermoFischerScientific | AB223652 | 1:100 |
| Mouse anti-AT8 | ThermoFischerScientific | AB223647 | 1:100 |
| Goat anti-Osteopontin | R&D systems | AF808 | 1:100 |
| Rat anti-CD31 | Dianova | DIA-310 | 1:100 |

**Supplementary Fig. 1 Histology staining in the hippocampus and thalamus of representative 18 month-old P301L mice. (**A, B) Hematoxylin & Eosin; (C, D) Prussian blue; (E, F) Alcian blue; (G, H) Periodic acid–Schiff. Scale bar 200μm.
