# Supplementary figures and images for: SWI and phase imaging reveal intracranial calcifications in the P301L mouse model of human tauopathy
Source: MAGMA. 2020 May 28;33(6):769–81. doi: 10.1007/s10334-020-00855-3 (PMC7669813; doi:10.1007/s10334-020-00855-3)

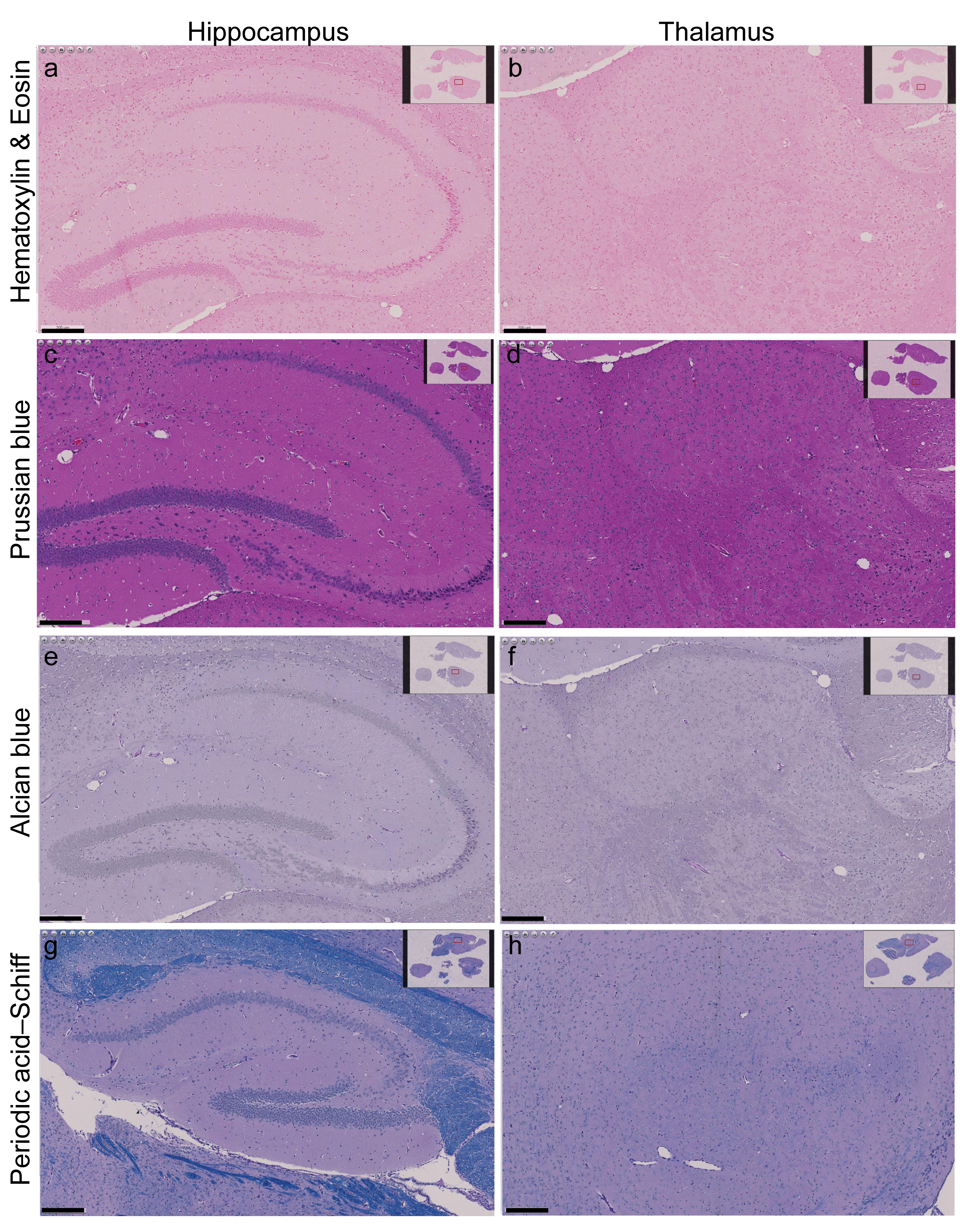

Supplement: Supplementary file 2 — Supplementary file2 (TIF 28324 kb) [file 10334_2020_855_MOESM2_ESM.tif]
